# Systematic Screening of Depression-Related Neurotoxicity Across 26 Bisphenols Reveals an ESR1–CREB1–GRIN2B-Associated Mechanism

**DOI:** 10.3390/ijms27167218

**Published:** 2026-08-13

**Authors:** Li Xie, Shanliang Yuan, Lu Gan, Rongheng Ma, Lei Tang, Qiang Xu, Weihong Li

**Affiliations:** 1 Basic Medical College, Chengdu University of Traditional Chinese Medicine, Chengdu 611137, China; 2 College of Information and Communication Engineering, Harbin Engineering University, Harbin 150001, China; 3 Key Laboratory of Mountain Surface Processes and Ecological Regulation, Institute of Mountain Hazards and Environment, University of Chinese Academy of Sciences, Beijing 100049, China

**Keywords:** bisphenols, depression, bisphenol A, machine learning, adverse outcome pathway, neurotoxicity

## Abstract

Bisphenols (BPs) are widespread environmental endocrine disruptors, but their potential depression-related neurotoxicity has not been systematically assessed. Here, we combined machine learning (ML), network toxicology, molecular docking, and in vivo experiments to screen 26 bisphenols for depression-related neurotoxic risk. We first integrated bisphenol A (BPA)-related targets with depression-related genes to build a chemical-gene-phenotype-disease network and an adverse outcome pathway (AOP) framework. We then used nine ML algorithms to rank the predicted risk of the 26 bisphenols. Molecular docking showed that the predicted high-risk compounds had strong binding affinity for estrogen receptor 1 (ESR1). The AOP analysis further suggested the involvement of the ESR1–cAMP response element-binding protein 1 (CREB1)–glutamate ionotropic receptor N-methyl-D-aspartate type subunit 2B (GRIN2B) pathway. In vivo experiments showed that BPA exposure caused neuronal damage in the cornu ammonis 3 (CA3) region of the rat hippocampus and significantly reduced the messenger RNA (mRNA) expression of the corresponding genes *Esr1*, *Creb1*, and *Grin2b* in hippocampal tissue. Overall, this study screened and prioritized the model-predicted depression-related neurotoxic risk of 26 bisphenols, supports a role for the ESR1–CREB1–GRIN2B pathway in BPA-induced neurotoxicity, and provides mechanistic evidence for bisphenol risk assessment.

## 1. Introduction

Bisphenol A (BPA), a representative bisphenol compound, is widely distributed in the global environment [1]. BPA is widely used in food contact materials, epoxy resins, and polycarbonate plastics [2]. As a result, humans are continuously exposed to this compound through various routes, including ingestion, inhalation, and skin contact [3]. Notably, there has been evidence that BPA can cross the blood–brain barrier and reach the brain tissue to destroy brain nerve function [4]. Consequently, the potential toxic effects of this chemical on the central nervous system (CNS) have become a hot topic in environmental health research [5].

Depression, as a representative mental illness, affects approximately 3% of the world’s population (about 264 million people) and has become a major global public health challenge [6]. Environmental pollutants may be a hidden driver of the increasing burden of depression [7]. Relevant evidence suggests that exposure to BPA induces anxiety in male mice by downregulating adrenergic receptors in the paraventricular nucleus, thereby triggering depression-like behavior [8]. Bisphenols (BPs), as ubiquitous environmental pollutants, may be associated with mental disorders such as depression, potentially by disrupting neural function and inducing neurotoxicity [9].

Considering the health risks of BPA, global regulatory restrictions have forced the industry to shift toward bisphenol analogs with structures similar to BPA, such as bisphenol C (BPC), bisphenol F (BPF) and bisphenol S (BPS) [10,11]. These compounds are commonly used in human daily necessities, such as epoxy adhesives, food can coatings, plastic products and so on [12,13]. However, this shift to “BPA-free” products has raised serious concerns about “regrettable substitution”. Current monitoring data indicate that emerging bisphenol compounds are frequently detected in environmental media such as soil and water, as well as in human urine and blood, with concentrations even exceeding those of BPA [14]. Because these analogs retain the two hydroxyphenyl functional groups in the bisphenol skeleton, they are very likely to exhibit similar or even stronger neurotoxicity than BPA [15,16].

However, the role of BPs as environmental triggers of neuropsychiatric disorders such as depression has not yet been systematically elucidated. As novel bisphenol derivatives continue to emerge, animal toxicity testing remains important for toxicological validation. At the same time, their long duration, high cost, and limited throughput point to the need for more efficient complementary approaches for early screening and risk prioritization. Machine learning (ML) algorithms, with their ability to handle complex nonlinear relationships, provide an efficient tool for screening the toxicity of environmental chemicals [17,18,19]. Data-driven models often face the “black box” challenge, lacking biological interpretation. Combining machine learning with the Adverse Outcome Pathway (AOP) framework may offer an effective strategy for improving predictive performance and mechanistic interpretation. AOP provides molecular mechanistic links between molecular initiating events (MIEs) triggered by chemicals and individual adverse outcomes (such as depressive behavior), facilitating the transition from “hazard identification” to “mechanistic understanding” [20].

Several studies have used machine learning and computational toxicology to predict the toxicity of bisphenol compounds. For example, machine learning has been applied to rank the hepatic toxicity of bisphenol analogs and to explore possible mechanisms using a computational adverse outcome pathway framework [21]. Other studies have predicted the bioconcentration factor and estrogen receptor bioactivity of BPA and its analogues [22]. Machine learning has also been combined with the Soil and Water Assessment Tool (SWAT) model to estimate the concentration, load, and ecological risk of bisphenol analogues at the river-basin scale [18], and used to assess the contribution of co-exposure to e-waste pollutants to human oxidative stress [19]. These studies have improved the rapid screening and risk assessment of bisphenol-related pollutants. However, most of them focused on hepatic toxicity, bioaccumulation, endocrine activity, environmental fate, or ecological risk. Depression-related neurotoxicity has received much less attention, and predicted risks have rarely been connected with interpretable toxicological pathways and in vivo evidence. Here, we focused on the depression-related neurotoxic risk of 26 bisphenols and combined machine learning, network toxicology, molecular docking, and animal experiments to investigate a potential estrogen receptor 1 (ESR1)–cAMP response element-binding protein 1 (CREB1)–glutamate ionotropic receptor N-methyl-D-aspartate type subunit 2B (GRIN2B) -associated mechanism.

This study focuses on 26 bisphenol analogs, combining nine machine learning algorithms with network toxicology analysis to predict their potential risk of inducing depression. Through molecular docking and enrichment analysis, we proposed a hypothetical AOP framework linking bisphenol exposure to depression-like neurotoxic outcomes, with a focus on the ESR1–CREB1–GRIN2B signaling pathway. This pathway was selected because ESR1 is a key target of bisphenol-related endocrine disruption, while CREB1 and GRIN2B are involved in synaptic plasticity and glutamatergic signaling, two processes closely related to depression. To validate this computationally derived hypothetical framework experimentally, in vivo animal studies using Sprague–Dawley (SD) rats were subsequently conducted. Behavioral assays were used to assess BPA-induced depression-like phenotypes, while morphological damage in hippocampal tissue and messenger RNA (mRNA) expression of key target genes were quantitatively analyzed. This integrated strategy combines high-throughput machine learning with in vivo validation, providing new evidence for understanding the contribution of environmental bisphenols to depression-related neurotoxicity.

## 2. Results

### 2.1. Bisphenol A (BPA)-Gene-Phenotype-Neurotoxicity Network

**Figure 1:**
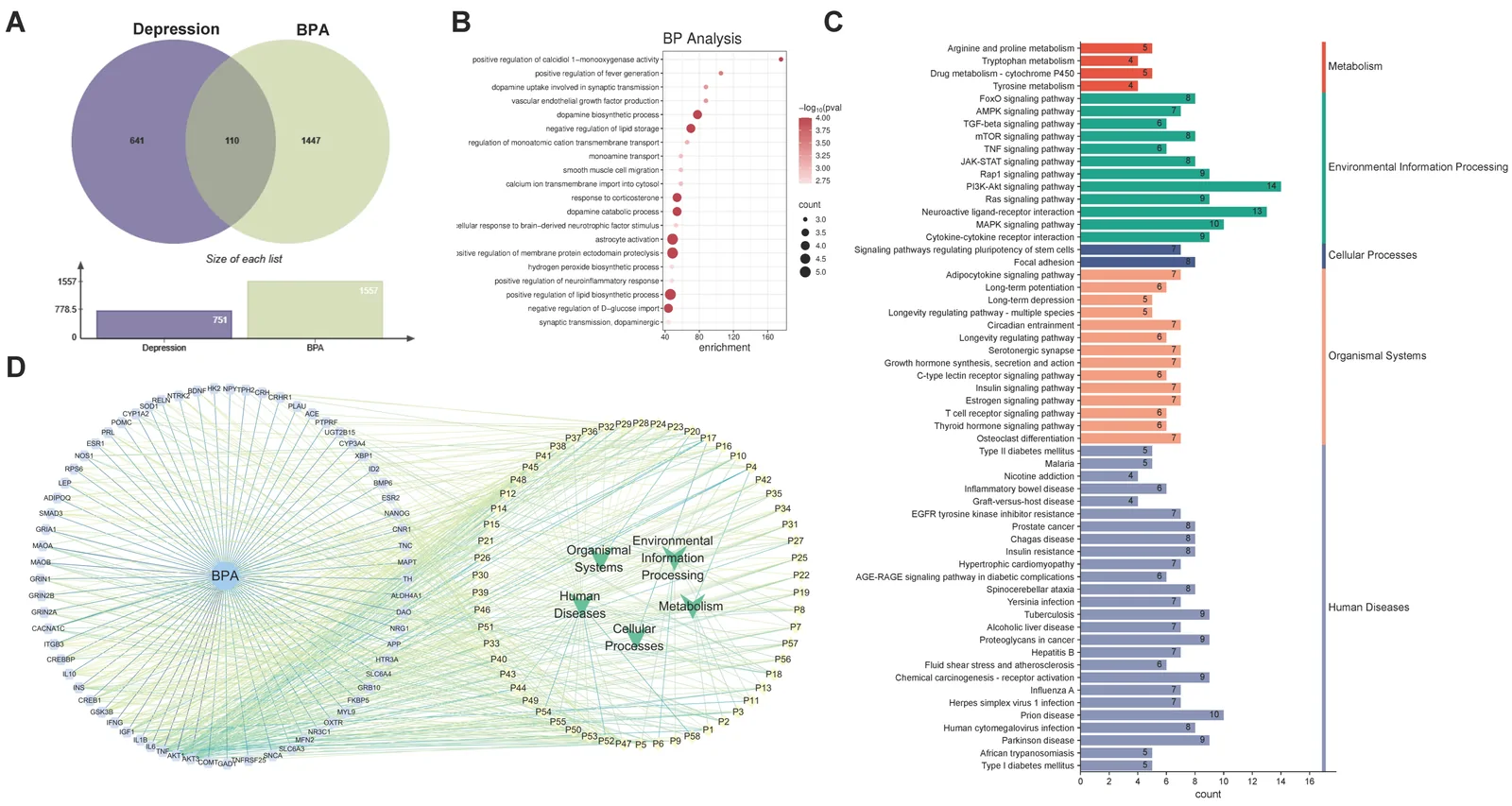
The chemical-gene-phenotype-depression network and phenotypes. (**A**) Venn diagram of genes from the BPA and depression datasets. (**B**,**C**) Phenotypes and KEGG pathways included in the target gene enrichment analysis. (**D**) Bisphenol A-gene-pathway-disease network. Blue nodes represent 69 genes, and yellow nodes represent 58 pathways. BPA, bisphenol A; KEGG, Kyoto Encyclopedia of Genes and Genomes.

We collected 1557 BPA-related targets, including 1218 from the Comparative Toxicogenomics Database (CTD), 87 from ToxCast, 205 from ChEMBL, 10 from STITCH, and 37 from SwissTargetPrediction, and 751 depression-related genes, including 655 from the Gene Expression Omnibus (GEO) and 104 from GeneCards. After taking the intersection of chemical substance-related genes and disease-related genes, we identified 110 common targets of BPA and depression (Figure 1A, Table S2). First, the 110 common targets were used for topological analysis, and topological analysis identified 69 core targets after excluding nodes that did not meet the predefined screening criteria.

Gene Ontology (GO) and Kyoto Encyclopedia of Genes and Genomes (KEGG) enrichment analysis was performed, and it was found that the main enrichment was related to the functions of Long-term potentiation, Long-term depression, Longevity regulating pathway-multiple species, Serotonergic synapse and other pathways, and dopamine uptake involved in synaptic transmission, positive regulation of neuroinflammatory response, dopamine biosynthetic process, synaptic transmission, dopaminergic and other biological processes (Figure 1B,C). Given the significant neurotoxicity associated with bisphenol A exposure, a topological analysis was performed on these 69 common target genes. The 69 common targets were used for topological analysis again. Cytoscape 3.8.2 was used to create and visualize the chemical-gene-phenotype-disease network (Figure 1D, Table S3). Based on the node_degree value ranking we identified 14 core targets: INS, AKT1, IL6, IGF1, CREB1, IL1B, LEP, APOE, IL10, TNF, ADIPOQ, ESR1, GRIN2B, IFNG as our research focus. These genes are used to construct the final computational adverse outcome pathway (cAOP) network.

### 2.2. Construction and Evaluation of Computational Adverse Outcome Pathway (cAOP) Network

The cAOP framework was created by manually selecting highly significant depressive phenotypes from the chemical-gene-pathway-disease network and linking them by hierarchy and biological linkage. Organisation for Economic Co-operation and Development (OECD) AOP handbook recommendations and literature evidence were used to evaluate BPA-ESR1-mediated AOP.

**Figure 2:**
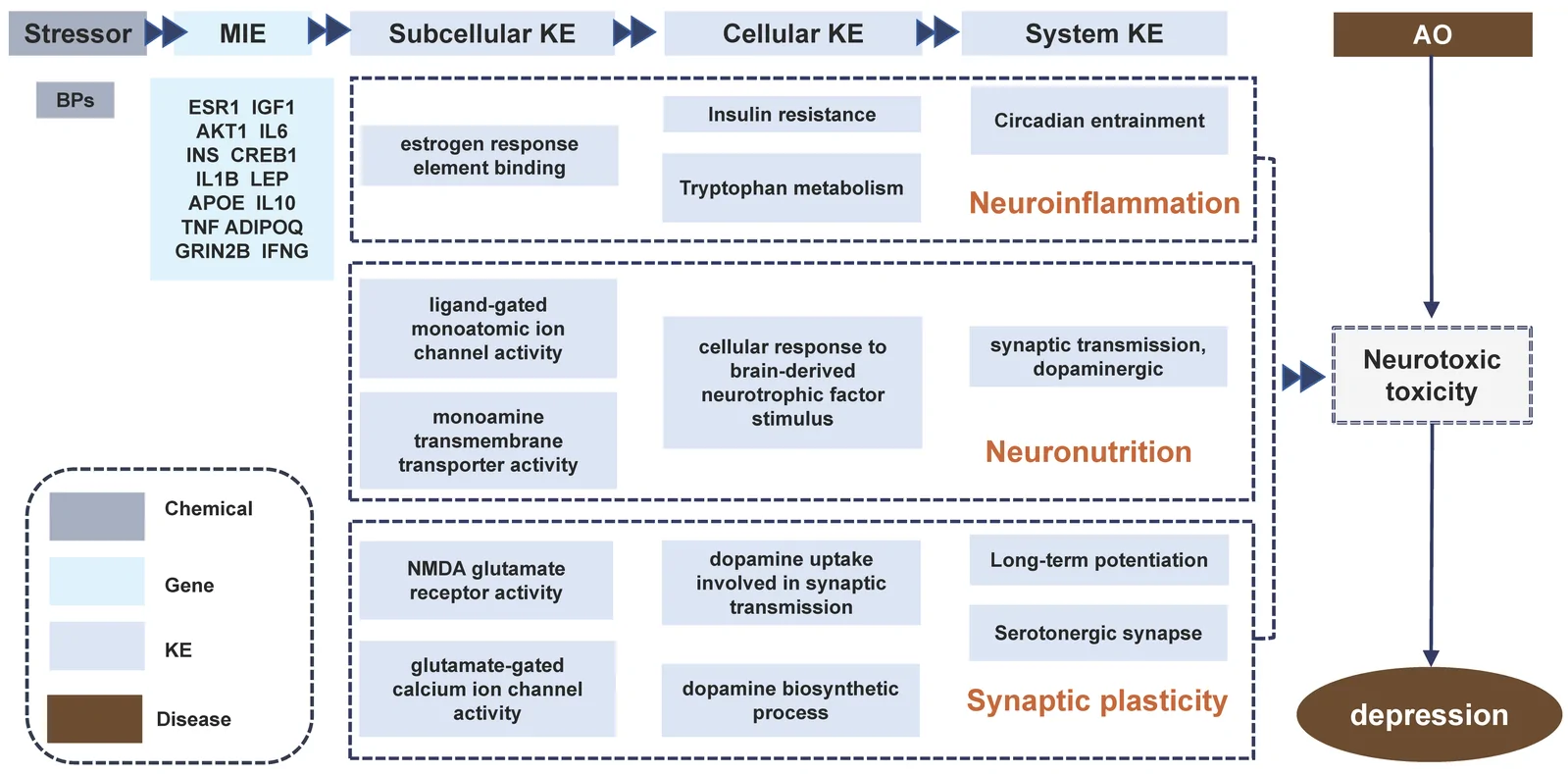
Computational adverse outcome pathway (cAOP) of BPA linked to Depression. The 15 key pathways and GO terms are subcellular, cellular, and systemic. BPs: Bisphenols; AO: Adverse Outcomes.

Based on enrichment analysis and biological correlations, we divided these target phenotypes and pathways into subcellular, cellular, and systemic levels. We noted that these characteristics exhibit a strong correlation with mental diseases, particularly depression, and these clear clues form the subsequent AOP network. According to the GO terms and KEGG pathways related to depression, we divided them into three categories (Figure 2).

The first group concerns neurotransmission and synaptic plasticity, including long-term potentiation, dopamine uptake, and the activity of ion channels that regulate postsynaptic membrane potential, among others. The second group mainly involves neuroinflammation and metabolic endocrine disorders, including Adipocytokine signaling pathway, Insulin resistance, estrogen response element binding. The third group mainly focuses on neurotrophic support and cell defense, including cellular responses to brain-derived neurotrophic factor (BDNF) stimulation, tau protein binding, Longevity regulating pathway-multiple species.

The framework integrates three key pathways of neurotransmission, neuroinflammation, and neurotrophic support, and systematically clarifies the complete mechanism of BPA from gene interference to depression phenotype. This integrated model reveals the complexity of bisphenol A-induced depression and provides a new perspective for analyzing its molecular events.

### 2.3. Machine Learning Models for Evaluating Depression Risk

To evaluate the potential of BPs to induce depression-related neurotoxicity, this study constructed 9 mainstream machine learning (ML) models for the 69 key target genes screened through gene network analysis. These models included Random Forest (RF), k-nearest neighbors (KNN), AdaBoost (Ada), Support Vector Machine (SVM), Linear Discriminant Analysis (LDA), Logistic Regression (LR), Decision Tree (DT), Naive Bayes (NB), and Multilayer Perceptron (MLP). Through the cross-combination of 69 targets and 9 algorithms, a total of 621 classification evaluation results were generated.

**Figure 3:**
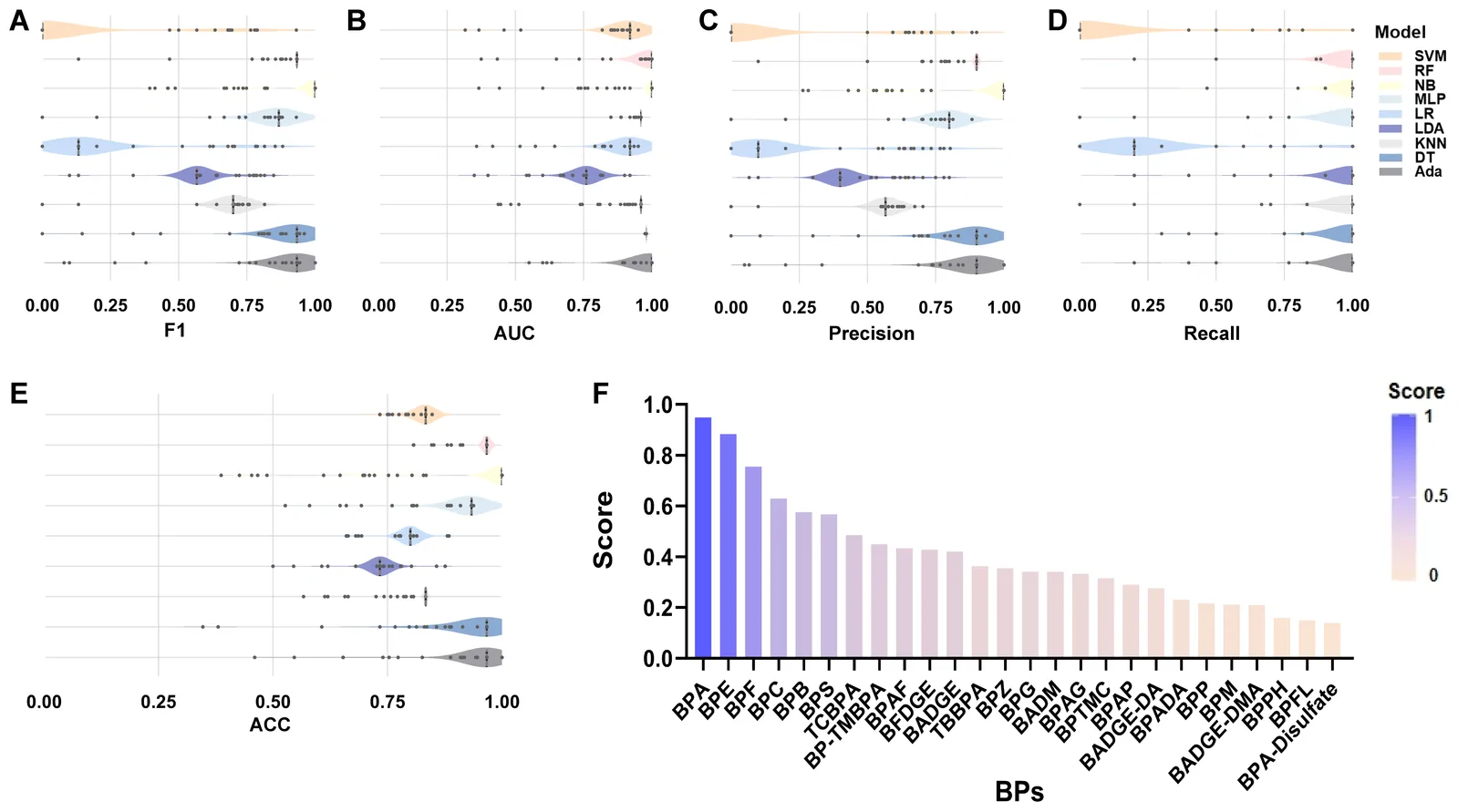
Using traditional machine learning models to predict depression risk (**A**–**E**) uses five metrics to evaluate the performance of machine learning models (F1, AUC, Precision, Recall, and ACC). (**F**) Predicting the depression risk scores of 26 BPs. AUC, area under the curve; ACC, accuracy; SVM, Support Vector Machine; RF, Random Forest; NB, Naive Bayes; MLP, Multilayer Perceptron; LR, Logistic Regression; LDA, Linear Discriminant Analysis; KNN, K-nearest Neighbors; DT, Decision Tree; Ada, AdaBoost; BPA, Bisphenol A; BPE, Bisphenol E; BPF, Bisphenol F; BPC, Bisphenol C; BPB, Bisphenol B; BPS, Bisphenol S; TCBPA, Tetrachlorobisphenol A; BP-TMBPA, Tetramethylbisphenol A; BPAF, Bisphenol AF; BFDGE, Bisphenol F diglycidyl ether; BADGE, Bisphenol A diglycidyl ether; TBBPA, Tetrabromobisphenol A; BPZ, Bisphenol Z; BPG, Bisphenol G; BADM, bisphenol A dimethacrylate; BPAG, bisphenol A glucuronide; BPTMC, Bisphenol TMC; BPAP, Bisphenol AP; BADGE-DA, Bisphenol A diglycidyl ether diacrylate; BPADA, 4,4′-(4,4′-Isopropylidenediphenoxy)diphthalic Anhydride; BPP, Bisphenol P; BPM, Bisphenol M; BADGE-DMA, Bisphenol A diglycidyl ether dimethacrylate; BPPH, Bisphenol PH; BPFL, Bisphenol FL; BPs, Bisphenols.

Figure 3A–E illustrates the performance metrics of nine machine learning models, including F1, area under the curve (AUC), precision, recall, and accuracy (ACC). The results (Figure 3A–E) show that, except for the logistic regression (LR) model, which exhibited relatively low and highly variable predictive performance across multiple metrics, the other eight machine learning models demonstrated good robustness and classification efficacy, with the median values of most metrics distributed between 0.75 and 1.00.

We scored the neurotoxicity (depression risk) of 26 typical BPs in the CompTox database. By converting the chemical structures into two-dimensional molecular descriptors and inputting them into the optimized model, the calculated scores reflect the probability of each BP member inducing depression-related neurotoxicity (Figure 3F). In order to compress the data range, the scores were displayed after log10(X + 1) transformation. The scoring results (Figure 3F and Table S4) indicate that there are certain differences in the depression risk scores of BPs with different structures. Among all 26 assessed BPs, BPA, bisphenol E (BPE), BPF, BPC, bisphenol B (BPB), and BPS were the top six compounds and were selected as prioritized high-risk candidates for subsequent analysis. BPA exhibited the highest predictive score (>0.9), indicating it has the strongest potential depression risk, followed closely by BPE, with its score trailing behind.

### 2.4. Molecular Docking Analysis

**Figure 4:**
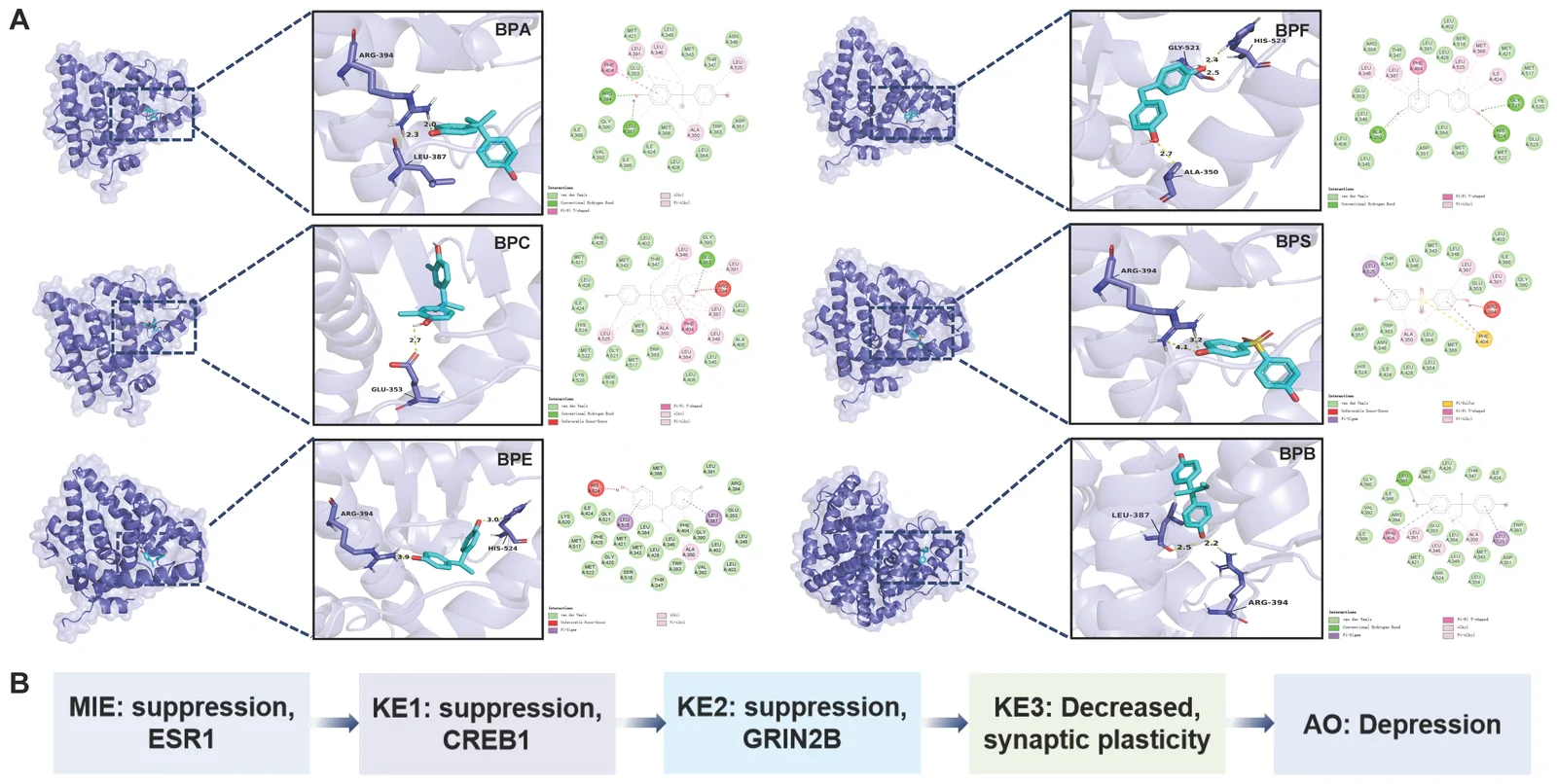
Molecular docking validation of BPs binding with core targets of depression. (**A**) Molecular docking validates the binding ability between BPs and ESR1. (**B**) The AOP framework mediated by ESR1. MIE: Molecular Initiating Event; KE, Key Event; AO, Adverse Outcome; BPA, Bisphenol A; BPB, Bisphenol B; BPC, Bisphenol C; BPE, Bisphenol E; BPF, Bisphenol F; BPS, Bisphenol S.

As shown in Figure 4A, all six BPs prioritized by the machine-learning ranking docked well into the ligand-binding domain of ESR1, with strong binding affinities ranging from −7.2 to −8.3 kcal/mol. BPC has the lowest binding energy (−8.3 kcal/mol); it forms conventional hydrogen bonds with GLU-353, and the binding is further stabilized by a PHE-404-mediated π-π T-shaped interaction and several hydrophobic interactions. The binding energies of BPB and BPA are −7.8 and −7.7 kcal/mol, respectively. BPB formed a conventional hydrogen bond with LEU-387, whereas BPA formed conventional hydrogen bonds with ARG-394 and LEU-387. BPE had a binding energy of −7.5 kcal/mol, showed no clear conventional hydrogen bond, and was mainly stabilized by hydrophobic interactions; HIS-524 was identified as an unfavorable donor–donor contact. BPF and BPS both have binding energies of −7.2 kcal/mol. BPF makes many hydrogen bonds with ALA-350, GLY-521 and HIS-524, whereas BPS creates an unfavourable donor-donor interaction with ARG-394.

### 2.5. Weight-of-Evidence (WoE) Assessment of Adverse Outcome Pathway (AOP) for BPA Linked to Depression

BPA is an endocrine disruptor that regulates human brain nerve function by binding to estrogen receptors. Therefore, ESR1 plays an important role in this process. To determine whether bisphenol A and ESR1 can bind, we conducted a molecular docking analysis to identify pockets in the protein structure that may interact with BPA.

AOP offers a structured way to link BPA exposure with downstream molecular and cellular events. In this study, the BPA–ESR1 interaction was defined as the putative molecular initiating event. Downregulation of CREB1 and GRIN2B was defined as key event 1 (KE1) and key event 2 (KE2), respectively, while impaired synaptic efficacy was defined as key event 3 (KE3) and linked to depression-like outcomes. Subsequently, we evaluated the WoE for the AOP using the guidelines from the OECD [23]. Based on existing epidemiological and experimental evidence, we assessed the strength of evidence for key events (KEs) and relationships between key events by considering biological plausibility, empirical support, and essentiality [24]; the evidence for each event or relationship is described as limited, moderate, or strong. Table 1, Table 2 and Tables S5–S9 summarize the importance of KEs and their related evidence. Overall, the AOP we propose—BPA–ESR1–CREB1–GRIN2B–synaptic dysfunction–depression-like outcomes—is supported by moderate to strong evidence, but further experimental validation is still needed.

### 2.6. BPA Exposure Induces a Depressive Phenotype in Rats

**Figure 5:**
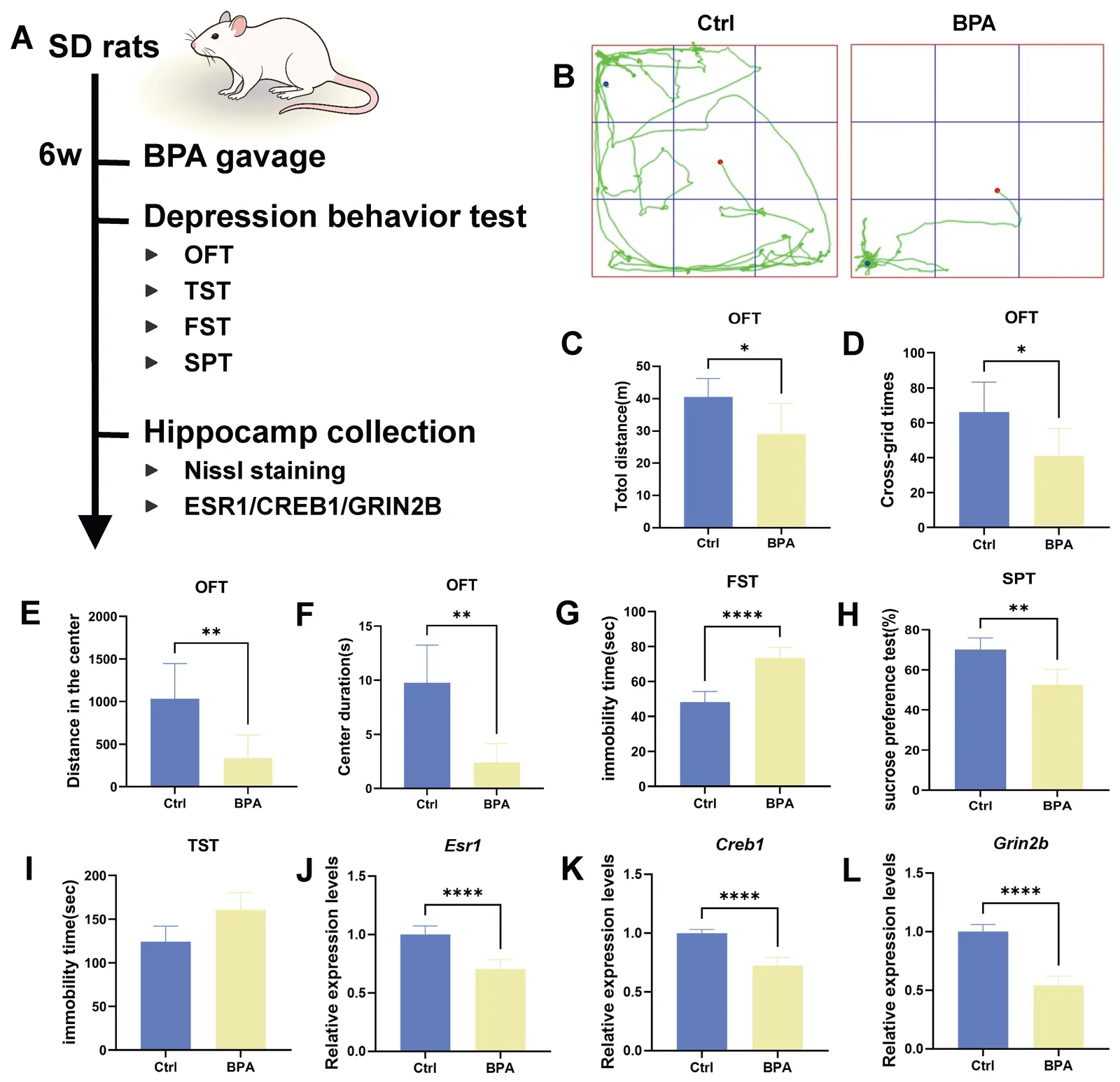
BPA induces depression in rats via the ESR1-CREB1-GRIN2B pathway. (**A**) Flowchart of the in vivo experiment. (**B**) Movement trajectories in the open field test (OFT), the green lines indicate the movement trajectories of rats in the open field. (**C**–**F**) Total distance traveled (**C**), number of crossings (**D**), distance traveled in the central area (**E**), and time spent in the central area (**F**) in the OFT. (**G**) Immobile time in the forced swim test (FST). (**H**) Sugar water preference rate in the sugar water preference test (SPT). (**I**) Immobile time in the tail suspension test (TST). (**J**–**L**) Relative mRNA expression levels of core target genes *Esr1* (**J**), *Creb1* (**K**), and *Grin2b* (**L**) were quantified by qPCR. Data are presented as mean ± standard deviation; *n* = 6 rats per group. * *p* < 0.05, ** *p* < 0.01, **** *p* < 0.0001 indicates a statistically significant difference compared with the control group. BPA, bisphenol A; SD, Sprague-Dawley; OFT, open field test; TST, tail suspension test; FST, forced swimming test; SPT, sucrose preference test; *Esr1*, estrogen receptor 1; *Creb1*, cAMP-responsive element-binding protein 1; *Grin2b*, glutamate ionotropic receptor NMDA type subunit 2B.

Open field test (OFT) monitoring revealed that animals treated with BPA exhibited a reduced overall range of movement (Figure 5A–E) and spent less time in the central area (Figure 5F), suggesting a decrease in exploratory behavior. In assessments of despair-related behaviors, the BPA group exhibited significantly prolonged immobility periods in the forced swim test (FST) (Figure 5G, *p* < 0.0001); similarly, the tail suspension test (TST) also revealed a comparable trend toward immobility (Figure 5I). Furthermore, BPA exposure impaired the animals’ reward-seeking behavior toward sucrose solution (Figure 5H, *p* < 0.01). To validate the key molecular mechanisms predicted by network toxicology and machine learning, mRNA expression levels of target genes (*Esr1*, *Creb1*, and *Grin2b*) in rat hippocampal tissue were assessed via quantitative real time PCR (qPCR) (Figure 5J–L). In the BPA group, mRNA expression of *Esr1* (Figure 5J), *Creb1* (Figure 5K), and *Grin2b* (Figure 5L) was significantly downregulated (*p* < 0.0001). These results suggest that BPA exposure induced depression-like behavior in a rat model, validating our hypothesis regarding the risk of BPA-induced depression.

### 2.7. BPA Exposure Induces Neuronal Damage in the Rat

**Figure 6:**
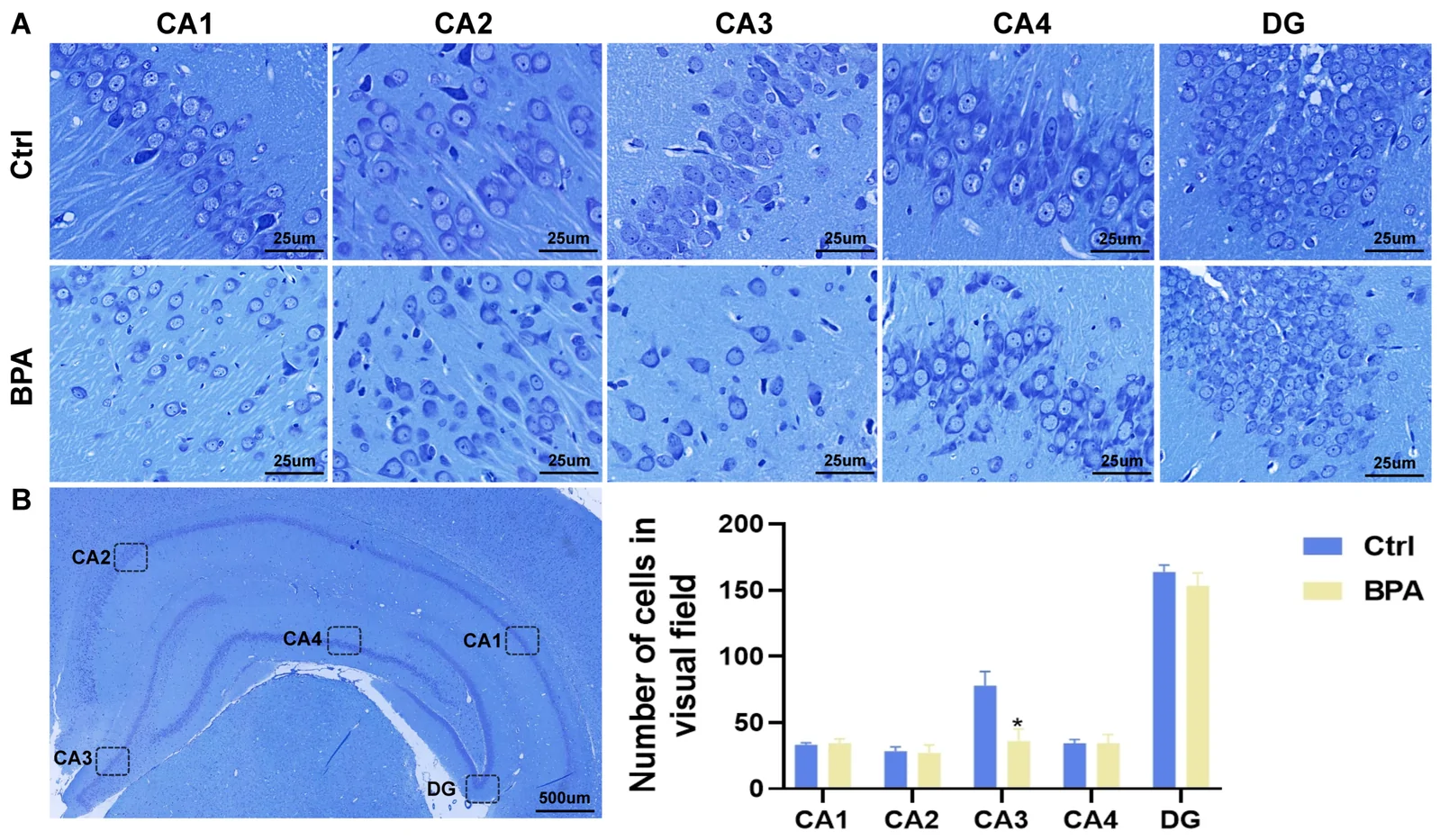
BPA exposure induces neuronal damage in the rat hippocampus. (**A**) Nissl stain images of different subregions (CA1, CA2, CA3, CA4, and DG) of the rat hippocampus in the control and BPA-exposed groups. Scale bar = 25 μm. (**B**) The left panel shows a panoramic microscopic image of rat hippocampal tissue; on the right are the results of Nissl body counts within the microscopic fields of view in each hippocampal subregion. Data are presented as mean ± standard deviation. *n* = 3 rats per group. * *p* < 0.05 indicates a statistically significant difference compared with the control group. BPA, bisphenol A; CA, Cornu Ammonis; Ctrl, control; DG, dentate gyrus.

To clarify the neurotoxic effects of BPA exposure, the morphology of neurons in five key anatomical regions of the hippocampus, including cornu ammonis (CA) 1 to CA4 and the dentate gyrus (DG) was examined using Nissl staining (Figure 6). As shown in Figure 6A, neurons in all hippocampal subregions of the control group were densely and regularly arranged; cell structures remained intact, and abundant, deeply stained Nissl bodies were visible around the cell nuclei. In contrast, neurons in the BPA group were loosely and irregularly arranged, accompanied by varying degrees of cellular atrophy and dissolution of Nissl bodies, indicating structural damage to the neurons. In contrast to the control group, BPA administration led to a decrease in Nissl bodies in the hippocampus, notably in the CA3 area (*p* < 0.05) (Figure 6B). These findings suggest that BPA exposure damages hippocampus neurons, with the CA3 region being more susceptible.

## 3. Discussion

In this study, we constructed an extensive computational approach that integrates machine learning and network toxicology to methodically evaluate the correlation between BPs and depression, as well as their neurotoxic potential. Although traditional toxicological studies have primarily focused on BPA, the neurotoxic characteristics of emerging BPs remain largely unexplored due to data scarcity. Our work fills this gap by successfully predicting the toxicity scores of 26 BPs and constructing a hypothetical AOP. Our findings support the hypothesis that BPA exposure may be a key environmental factor contributing to depression-related neurotoxicity and suggest the existence of a core regulatory network involving ESR1, CREB1, and GRIN2B.

Depression is a complex heterogeneous disease influenced by environmental factors. Although epidemiological studies suggest an association between BPA and depression [38], its potential mechanisms remain elusive, and data on emerging BPs are even scarcer [39]. Our research provides critical evidence to fill this knowledge gap by identifying the neurotoxic potential of emerging BPs through nine machine learning algorithms. By highlighting the structural and functional similarities between emerging BPs and BPA, our results suggest that the risk of depression may not be limited to BPA but also extends to its widely used substitutes, challenging the safety of these so-called “BPA-free” alternatives.

AOP is playing an increasingly crucial role in modern risk assessment [40]. We propose a computational AOP (cAOP) framework that spans subcellular, cellular, and system levels. We integrated a “chemical-gene-disease” network and hypothesized that the binding of BPs to nuclear receptors triggers a series of KEs. These events include the disruption of dopaminergic synaptic transmission and the inhibition of LTP. Cellular disruptions cause structural plasticity impairments in the hippocampus and prefrontal cortex, which lead to depression. This framework supports an increasingly growing consensus: controlling environmental exposure is a crucial yet often overlooked aspect of mental health prevention.

This study identifies the interaction between BPA and ESR1 as a putative molecular initiating event and identifies CREB1 as a key event in BPA-induced depression-like neurotoxicity. Although the role of BPA as an endocrine disruptor is well-known, our results specifically link its anti-estrogenic activity to neurobehavioral outcomes. The estrogen signaling mediated by ESR1 is crucial for neuroprotection and emotional regulation. As emphasized by our Weight of Evidence (WoE) assessment, the expression of ESR1 in gamma-aminobutyric acid (GABA)-ergic neurons is crucial for emotional regulation, and its polymorphism is significantly associated with susceptibility to depression. By interfering with ESR1 expression, BPA may mimic a state of estrogen deficiency in the brain, which explains why behavioral changes associated with BPA exposure are similar to those of perimenopausal depression.

Our cAOP specifically elucidates the ESR1-CREB1-GRIN2B axis. First, we identified CREB1 as KE1. Current research indicates that estrogen signaling can modulate the phosphorylation and activity of CREB1 via the phosphoinositide 3-kinase/protein kinase B (PI3K/AKT) pathway [33]. BPA-induced ESR1 inhibition results in the suppression of downstream CREB1 activity. As a major regulatory factor of BDNF, the downregulation of CREB1 is a consistent finding in postmortem brain tissue of patients with depression [34]. Secondly, this pathway extends to KE2: the downregulation of GRIN2B (which encodes the NMDA receptor GluN2B subunit), a gene transcriptionally regulated by CREB1. Finally, changes in NMDA receptor composition lead to impaired synaptic plasticity (KE3)—manifested as weakened LTP and synaptic atrophy—this is the final physiological impairment before the onset of depressive behavior [35].

Molecular docking provides structural validation that further strengthens this hypothesis. Our analysis shows that high-risk BPs can form stable complexes with the ESR1 ligand-binding domain, providing a structural basis for potential interaction with ESR1. Importantly, Compounds predicted to have a higher potential for neurotoxicity also tend to exhibit more favorable binding affinity for ESR1. This indicates that the affinity of BPs for ESR1 is a key structural determinant of their neurotoxic potential. The binding energies of the six BPs with ESR1 differ slightly, probably because they have similar biphenyl scaffolds. The common structure enables the formation of similar hydrogen bonds in the ESR1 binding pocket; thus, hydrogen bonding is not likely to be the main reason for the minor differences in the docking scores. Conversely, their different substituents may interact differently with residues in the hydrophobic pocket, such as LEU, ALA, PHE, and MET. Therefore, these hydrophobic interactions are weak, but may account for the subtle differences in binding energies for these BPs.

Combining machine learning with AOP has proven to be a powerful strategy for predictive toxicology. Given the rapid emergence of new BPs, traditional toxicity testing is often too slow and expensive. Our research highlights the practicality of machine learning algorithms in high-throughput toxicity prediction. Using two-dimensional molecular descriptors, our models, such as RF and SVM, effectively scored the neurotoxicity of 26 BPs. It is worth noting that we observed that structural analogs such as BPE and BPF also received high risk scores, which corroborates previous research findings. For example, studies have shown that more than half of the tested BPA analogs (9 types) exhibited stronger cytotoxicity than BPA [14]. This approach, which combines machine learning with network analysis, provides interpretability, revealing not only the toxicity of chemicals but also how their structural features interact through specific biological networks to induce disease phenotypes such as depression.

Our in vivo rat experiments demonstrated that BPA exposure induced core symptoms of depression: namely, reduced exploratory behavior, anhedonia, and behavioral despair. At the histological level, significant damage and loss of neurons in the hippocampal CA3 region, as observed via Nissl staining, provided direct pathological evidence of BPA-mediated central nervous system toxicity. More importantly, according to the qPCR results, following exposure to BPA, the expression levels of the *Esr1*, *Creb1*, and *Grin2b* genes in the hippocampus of rats were significantly reduced. This in vivo molecular profile aligns with our cAOP framework, which posits that BPA, as an environmental endocrine disruptor, may interfere with ESR1-associated signaling, thereby cascading to block downstream CREB1/GRIN2B signaling essential for maintaining synaptic plasticity and neuronal survival.

Our findings are consistent with previous evidence that bisphenol exposure can affect mood-related behaviors and neurofunction. Previous epidemiological studies have examined the relationship between serum BPAF levels and depressive symptoms in adolescents [7]. Animal studies have also shown that exposure to BPA or BPS can induce depression- or anxiety-like behaviors and affect the medial prefrontal cortex, the basolateral amygdala, and the serotonergic system [38,41,42]. These findings support a link between bisphenols and depression-related neurotoxicity. However, most previous studies focused on BPA or only a few substitutes, and their conclusions may differ depending on sex, exposure window, behavioral endpoint, and brain region. Our study extends this work by comparing 26 bisphenols within the same depression-related toxicological framework.

Our results also reinforce the concern that “BPA-free” substitutes are not necessarily safer. Singh et al. reported that BPS and BPF can induce neurobehavioral abnormalities, inflammatory changes, and neurotransmitter disturbances [43]. Another study showed that several BPA analogues may exhibit stronger cytotoxicity, endocrine-disrupting activity, neurotoxicity, or aryl hydrocarbon receptor (AhR) activity than BPA [15]. Consistent with these reports, our machine-learning results showed that, besides BPA, substitutes such as BPE and BPS also had high predicted depression-related neurotoxic potential. This indicates that BPA alternatives still require systematic evaluation and cannot be assumed to be safe simply because they are used as substitutes for BPA. At the mechanistic level, previous research on depression has linked hippocampal synaptic dysfunction, oxidative stress, BDNF-related signaling changes, and microglia-mediated synaptic pruning to depression-like phenotypes [44,45,46,47]. Meng et al. also found that low-dose bisphenol exposure can impair hippocampal neuronal development and disrupt the glutamate/GABA balance [48]. Building on these findings, the present study further revealed that BPA exposure causes neuronal damage in the CA3 region of the rat hippocampus, accompanied by decreased expression of *Esr1*, *Creb1*, and *Grin2b*.

Our study has inherent limitations in computational methods. First, predictive models rely on existing databases, which may have biases against compounds like BPA that are well-studied. It should be noted that the 69 core targets in this study were derived from the overlap between BPA-related targets and depression-related genes. Thus, the top ranking of BPA was partly expected and should not be viewed as a fully independent or unbiased comparison of risk across all bisphenols. In this study, BPA served mainly as a reference compound to define a depression-related toxicological space. Within this framework, the key finding is not simply that BPA ranked highest, but that several BPA alternatives, such as BPE and BPS, also showed high depression-related neurotoxic potential. These results suggest that some emerging bisphenol substitutes may share BPA-like neurotoxic risks and should be tested further.

Second, although we established an in vivo rat model, the limited similarity between this model and human conditions should be considered. The results may not fully reflect biological responses in the human body. Rat models cannot fully replicate the complex pathophysiological processes of human depression, nor can they reflect real human exposure scenarios, such as long-term, low-dose, and mixed exposure. Therefore, extrapolation of these findings to humans should be made with caution. In addition, long-term epidemiological longitudinal cohort studies are still needed to confirm the causal relationship between exposure to BPs and depression in real-world populations.

Third, our model primarily focuses on the effects of single chemicals, whereas real-world exposures often involve complex mixtures. Although this study has successfully established a rat model and conducted in vivo validation of the depressogenic risk associated with BPA exposure and its core target (the ESR1 axis), future toxicological validation should be further expanded to include other novel bisphenol analogs identified as high-risk by our machine learning model (e.g., BPE and BPS).

## 4. Materials and Methods

### 4.1. Collection of Targets for Depression and BPA

**Figure 7:**
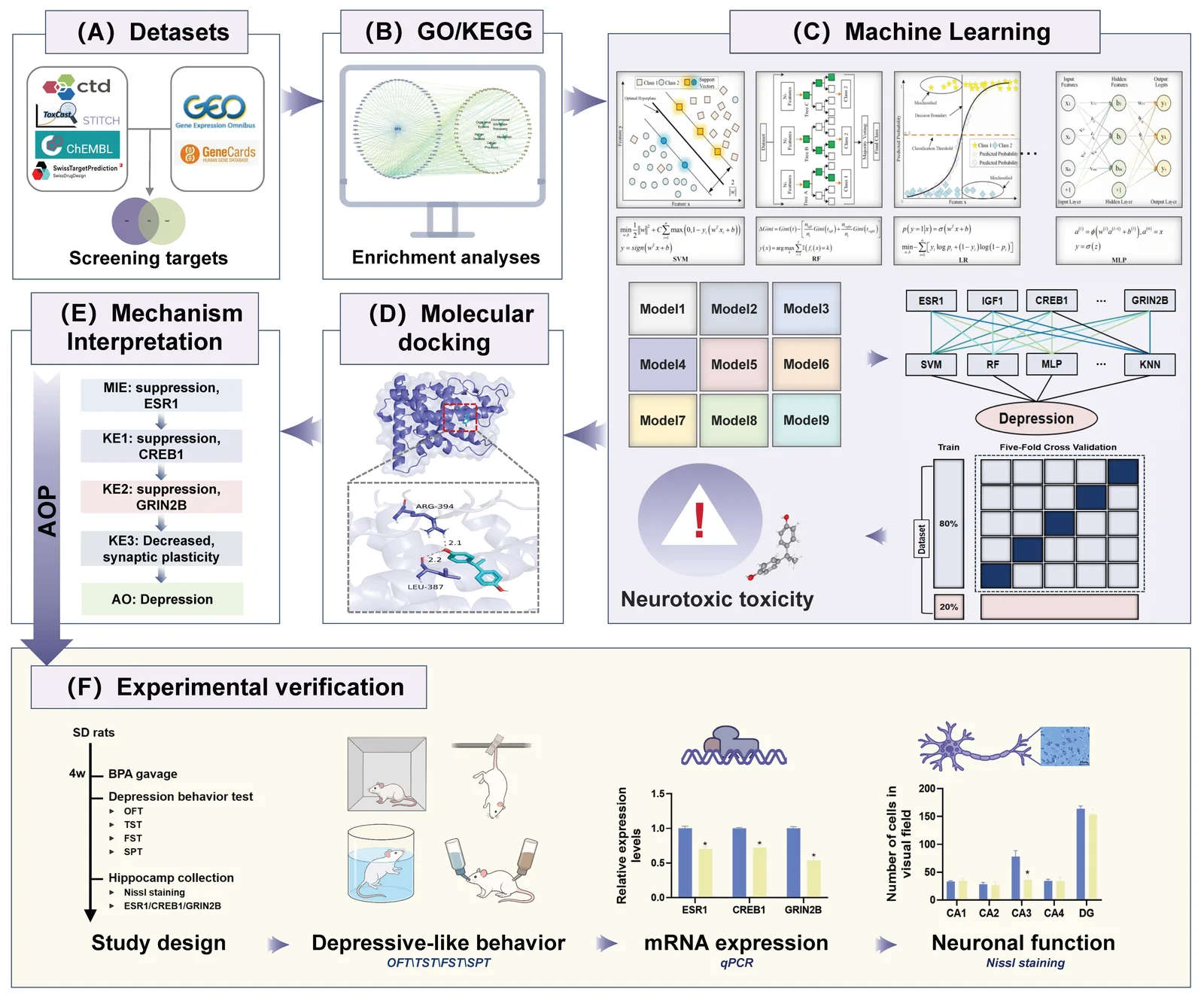
Workflow framework combining network toxicology, machine learning, and in vivo experiments. (**A**) Retrieval of gene sets from multiple public databases. (**B**) GO and KEGG functional enrichment analysis and visualization. (**C**) Construction of machine learning models for predicting depression risk and neurotoxicity, with the dataset validated using five-fold cross-validation. (**D**) Virtual validation via molecular docking of core target proteins and BPs. (**E**) Establishment of an AOP framework from MIE to AO. (**F**) In vivo experimental protocol to validate BPA exposure-induced depression-like behavior and neurotoxicity in rats. * *p* < 0.05. GO, Gene Ontology; KEGG, Kyoto Encyclopedia of Genes and Genomes; SVM, Support Vector Machine; RF, Random Forest; LR, Logistic Regression; MLP, Multilayer Perceptron; KNN, K-nearest Neighbors; AOP, Adverse Outcome Pathway; MIE, Molecular Initiating Event; KE, Key Event; AO, Adverse Outcome; ESR1, Estrogen Receptor 1; CREB1, cAMP Responsive Element Binding Protein 1; GRIN2B, Glutamate Ionotropic Receptor NMDA Type Subunit 2B; SD, Sprague–Dawley; OFT, Open Field Test; TST, Tail Suspension Test; FST, Forced Swimming Test; SPT, Sucrose Preference Test; qPCR, quantitative polymerase chain reaction; CA, Cornu Ammonis; DG, Dentate Gyrus.

Figure 7 illustrates the workflow of this framework. The workflow consists of several stages, including data collection, machine learning model predictions, molecular docking, and validation through animal experiments. The target set of depression was mainly constructed using GEO (https://www.ncbi.nlm.nih.gov/geo/ (accessed on 20 February 2026)) and GeneCards databases (https://www.genecards.org/ (accessed on 20 February 2026)). The keywords used in these databases include “Depression” and “Major depressive disorder”. After data integration and removal of duplicate data, a database of depression targets was established. To identify BPA-related targets, chemical structures and Simplified Molecular Input Line Entry System (SMILES) strings were retrieved from PubChem (https://pubchem.ncbi.nlm.nih.gov/ (accessed on 21 February 2026)). The SMILES strings were then used to search ChEMBL (https://www.ebi.ac.uk/chembl/g/ (accessed on 25 February 2026)), with the target species restricted to *Homo sapiens*. The SMILES notation is also used to input and retrieve targets from the STITCH (http://stitch.embl.de/ (accessed on 26 February 2026)) and SwissTargetPrediction databases (https://swisstargetprediction.ch/ (accessed on 26 February 2026)). In addition, BPA-related genetic data were retrieved from CTD (https://ctdbase.org (accessed on 27 February 2026)) and ToxCast (https://www.epa.gov/comptox-tools/toxicity-forecasting-toxcast (accessed on 27 February 2026)). After removing duplicate targets, a complete list of bisphenol A targets was obtained.

### 4.2. Construction of a Compound-Gene-Phenotype-Disease Network

By taking the intersection of BPA and depression targets, we identified potential targets representing BPA-induced depression. The analysis of interactions between these genes was performed in STRING (https://string-db.org/ (accessed on 1 March 2026)). The resultant data were then exported to Cytoscape 3.8.2 software for topological analysis, where the essential topological characteristics of each node (denoting protein) and edge (indicating interaction) were computed, encompassing degree centrality, betweenness centrality, and closeness centrality. The DAVID database (https://davidbioinformatics.nih.gov/ (accessed on 3 March 2026)) was used for GO and KEGG functional annotation. The annotated phenotypes obtained from the topological analysis were further screened to retain those most relevant to the objectives of this study. Finally, the chemical-gene-pathway-disease network was constructed using Cytoscape 3.8.2 software.

### 4.3. Machine Learning Models

We used nine machine learning algorithms to assess the depression-related neurotoxicity potential of BPs, including LR, RF, KNN, SVM, LDA, DT, NB, Ada, and MLP. The RDKit package (version 2023.09.5) was used to convert the chemical structures of BPs into two-dimensional molecular descriptors. The molecular features of each chemical substance and the activation status (“0” indicates inactive, and “1” indicates active) were used as the input data for model building. Invalid molecular descriptors were filtered out during the conversion of SMILES strings to molecular descriptors. The evaluation metrics included accuracy (ACC), F1-score, recall, precision, and area under the curve (AUC). The specific calculation formulas are listed below. For the basic variables used in the formulas, TP, TN, FP, and FN are defined as the frequencies of true positives, true negatives, false positives, and false negatives, respectively.

(1) $$\mathrm{Accuracy}=\frac{\left(TP+TN\right)}{\left(TP+TN+FP+FN\right)}$$

(2) $$F1-\mathrm{Score}=2\cdot \frac{TP}{FP+2TP+FN}$$

(3) $$\mathrm{Recall}=\frac{TP}{\left(TP+FN\right)}$$

(4) $$\mathrm{Precision}=\frac{TP}{\left(TP+FP\right)}$$

(5) $$AUC=\int_{0}^{1}t_{pr}{(f}_{pr})df_{pr}=P(X_{p}>X_{n})$$

To quantify the depression-related neurotoxic potential of each bisphenol compound, this study constructed an integrated toxicity score based on machine learning prediction results and the association scores between target genes and depression. To avoid the influence of differences in the scale of gene scores, the raw gene scores were first normalized using min–max normalization, so that the values of all genes ranged from 0 to 1. Subsequently, an oversampling technique was applied to expand the minority-class samples, thereby reducing the impact of class imbalance. Next, for each bisphenol compound, the trained machine learning models were used to predict whether the compound could potentially act on or activate depression-related neurotoxic targets. The toxicity score was calculated by the following formula.

(6) $$\mathrm{Risk Score}=\sum_{i=1}^{n}r_{i}*g_{i}$$

In this formula, *n* represents the number of genes; *r_i_* represents the model’s predicted result; *r_i_* = 1 indicates that the model predicted the compound to be active against the corresponding target, whereas *r_i_* = 0 indicates that the compound was predicted to be inactive. *g_i_* represents the gene score. When the model was able to output probability values, the predicted probability of the active class was used as *r_i_*, so that the score reflected not only whether the compound was active, but also the confidence level of the model prediction. A higher score indicated a higher model-predicted risk for depression-related neurotoxicity.

### 4.4. Identification and Evaluation of Computational Adverse Outcome Pathway (cAOP) Networks

Based on topological analysis of phenotypic networks and supporting literature evidence, highly significant depression-related phenotypes were identified. These phenotypes are then interconnected based on their levels and biological relationships, establishing a cAOP framework at the subcellular, cellular, and system levels [49]. AOPs were evaluated using the WoE approach, which assessed the biological plausibility and empirical support of KERs, the necessity of KEs, species consistency, and chemical applicability [50].

### 4.5. Molecular Docking Analysis

The crystal structure of ESR1 was obtained from the Protein Data Bank under PDB ID 9BQE (https://www.rcsb.org/ (accessed on 14 March 2026)). The 3D structures of the bisphenol compounds were downloaded from PubChem (https://pubchem.ncbi.nlm.nih.gov/ (accessed on 14 March 2026)). AutoDockTools 1.5.7 was used to prepare the receptor and ligands by removing water molecules and unrelated small molecules, adding hydrogens, assigning Gasteiger charges, and converting the structures into pdbqt format. Molecular docking was performed with AutoDock Vina 1.2.5, and binding affinities were calculated from the best-ranked docking poses. For protocol validation, the co-crystallized ligand was extracted from the 9BQE structure and re-docked into its original binding pocket. Before redocking, the ESR1 receptor was prepared by removing the ligand, adding hydrogens, repairing residues, and optimizing the structure. The re-docked pose was compared with the original crystallographic pose in PyMOL 2.6.0, and the root-mean-square deviation (RMSD) was calculated. The 3D binding modes were visualized with PyMOL 2.6.0, and the 2D interaction diagrams were generated with Discovery Studio 2019.

### 4.6. Experimental Animals and Study Design

This study used 18 male SD rats (SPF Biotechnology, Beijing, China) aged 6 weeks and weighing approximately 180 g. All experimental animals were housed at the Laboratory Animal Center of Chengdu University of Traditional Chinese Medicine for routine care. The environmental parameters for animal housing were set as follows: relative humidity between 50% and 60%, room temperature maintained at 25 ± 5 °C, and a 12 h light/12 h dark lighting cycle. Following a standard acclimatization period, all rats were randomly divided into a control group and a BPA group (9 rats per group). The control group received 0.5 mL corn oil by oral gavage, whereas the BPA group received BPA (Aladdin, Shanghai, China) dissolved in corn oil (Meilunbio, Dalian, China) at 50 μg/kg/day for 6 weeks [51]. The selection of BPA doses was based on the low-dose regimens commonly used in neurobehavioral studies of rodents and the range of average bisphenol A exposure levels among adults in North America [52,53,54].

All animal procedures involved in this study were approved in advance by the Animal Ethics Committee of Chengdu University of Traditional Chinese Medicine. The entire study protocol fully complies with the National Guidelines for the Welfare and Management of Laboratory Animals issued by the Ministry of Science and Technology of China. For seven days prior to the start of the formal intervention, the experimental rats were allowed to eat and drink ad libitum. This study adhered to the established standards. Throughout all stages of the experiment, researchers have taken every possible precaution to minimize discomfort and pain for the laboratory animals.

### 4.7. Behavioral Testing

#### 4.7.1. Sucrose Preference Test

The SPT protocol was adapted from the method adopted by Li et al. [55]. First, the animals were housed individually in cages, each equipped with two water bottles containing 1% sucrose solution, for a full day (24 h). A bottle of filtered water was added on the second day, and the bottles were switched within 24 h. After fasting and water deprivation for 24 h, a 12 h test was conducted. The final preference index was calculated using a specific ratio: the mass of sucrose solution consumed divided by the total mass of fluid (the sum of purified water and sucrose solution) consumed.

#### 4.7.2. Open Field Test

To assess whether BPA affects spontaneous motor activity in rats, the open field test (OFT) was employed for analysis in this study. Each rat was placed in a completely black box to explore freely for 5 min. VisuTrack animal behavior analysis software (version 2.0; Xinruan, Shanghai, China) was used to record the following metrics: Total distance, number of grid crossings, distance travelled in the central area, and time spent in the central area.

#### 4.7.3. Tail Suspension Test and Forced Swim Test

The TST and FST were commonly employed to evaluate depression-like behaviour in animals. In the TST, a rat’s tail was affixed with tape to suspend it 20 cm above the ground, and the duration of immobility was measured over a period of 6 min. In the FST, rats were in a 20-cm-diameter, 60-cm-tall transparent glass cylinder filled with 23 °C water. The rats were placed inside the cylinder, and the total immobility time over 6 min was recorded.

### 4.8. Collection of Brain Tissue

On the final day of the six-week BPA intervention, the rats were fasted for 24 h; this pre-sampling fasting period did not result in a significant decrease in body weight or any observable adverse effects. During the experiment, to minimize distress to the rats, they were euthanized by cervical dislocation under light anesthesia with sodium pentobarbital (Sinopharm, Shanghai, China). Six rats were randomly selected from each group for rapid brain tissue collection. After cold washing with saline, the hippocampus was dissected and used for qPCR analysis; All brain samples were kept at −80 °C until analysis. The remaining three rats in each group were euthanized for histopathological examination; following intracardiac perfusion with cold saline, the brains were immediately fixed in 4% paraformaldehyde (Servicebio, Wuhan, China).

### 4.9. Nissl Staining

Hippocampal tissue was fixed in 4% paraformaldehyde (Servicebio, Wuhan, China), dehydrated, embedded in paraffin, and sectioned into 5-μm-thick sections. The sections were incubated in Nissl stain (Toluidine Blue Method; Cat. No. G1436; Solarbio, Beijing, China) in an incubator (GSP-9160MBE, Boxun, Shanghai, China) at 50–60 °C for 40 min, followed by a brief wash with distilled water and rapid decolorization in 95% ethanol. They were dehydrated in anhydrous ethanol and clarified in xylene, and affixed using a neutral resin. They were examined and photographed using a light microscope (DM6 B, Leica Microsystems, Wetzlar, Germany): Nissl bodies appeared blue-violet, cell nuclei appeared pale blue-violet, and the hippocampus region was examined to assess morphological changes in neurons.

### 4.10. Messenger RNA (mRNA) Expression Determined by Quantitative Real-Time PCR (qPCR)

Total RNA was isolated using the RNA Isolater Total RNA Extraction Reagent (Vazyme, Nanjing, China). RNA concentration and purity were measured by NanoVue Plus micro-spectrophotometers (Biochrom Ltd., Cambridge, UK). The extracted RNA was reverse-transcribed into complementary DNA (cDNA) by the HiScript III All-in-one RT SuperMix Perfect for qPCR Kit (Vazyme, Nanjing, China). The primers for *Esr1*, *Creb1*, and *Grin2b* were designed and synthesised by TaKaRa (Dalian, China) (Table S1). Real-time quantitative PCR was conducted utilising the Bio-Rad CFX Real-Time PCR System (Bio-Rad, Hercules, CA, USA). The reaction conditions included a 5 min pre-denaturation at 95 °C, succeeded by 40 cycles comprising 10 s of denaturation at 95 °C and 30 s of annealing at 60 °C. *Gapdh* served as the internal reference gene, and relative mRNA expression levels were determined using the 2^−ΔΔCt^ technique.

## 5. Conclusions

This study employed an innovative computational strategy to successfully predict the depression-related neurotoxicity risks of 26 bisphenol compounds. Using the AOP framework and in vivo experiments, it suggests that BPA may damage neurons via the ESR1–CREB1–GRIN2B pathway, thereby promoting depression-like neurotoxicity. These findings deepen our understanding of the neurotoxic effects of environmental pollutants and provide a basis for assessing the health risks of BPA alternatives and developing regulatory strategies for emerging environmental pollutants.

## Figures and Tables

**Table 1:** Evaluation of the Evidence of KEs.

Events	Evidence	WoE Support
MIE: suppression ESR1	ESR1 has been identified as a key target linking BPA exposure with major depressive disorder in network-based analyses [25]. Benzophenone-3 lowers ESR1 mRNA and protein expression in the neocortex, causing apoptosis and neurotoxicity [26].	Moderate
KE1: suppression CREB1	Depression lowers CREB1 expression, and its protein directly interacts with mental disease risk genes [27,28].	Moderate
KE2: decrease GRIN2B	The decrease in GRIN2B expression is closely related to depression and synaptic plasticity disorder [29,30].	Moderate to strong
KE3: Synaptic plasticity	Depression patients’ cognitive and emotional symptoms are linked to synaptic loss, which weakens long-term potentiation (LTP) in the hippocampus and strengthens long-term depression (LTD) [31,32].	strong

**Table 2:** Evaluation of the Evidence of Key Event Relationships (KERs).

Events	Evidence	WoE Support
MIE to KE1, suppression of ESR1, led to suppression of CREB1	ESR1 can regulate PI3K/AKT/CREB signal. In the experimental model, pharmacological inhibition of ESR1 blocked the activation of CREB pathway [33].	Moderate
KE1 to KE2, suppression CREB1, led to decreased GRIN2B	CREB1 regulates activity-dependent transcription and plasticity-related genes, BDNF/TrkB signal supports N-methyl-d-aspartate receptor(NMDAR) with Glu2B in synapse, CREB1 inhibition may indirectly lead to GluN2B/NMDAR dysfunction [34,35].	Moderate
KE2 to KE3, decreased GRIN2B, led to impaired synaptic plasticity.	GRIN2B mutation is related to decreased NMDAR current and impaired synaptic plasticity in hippocampus [36].	strong
KE3 to AO, synaptic plasticity impairment, led to depression.	Changes in synaptic plasticity are intimately linked to stress-induced depressive-like behavior [37].	strong

## Data Availability

The original contributions presented in this study are included in the article/Supplementary Materials. Further inquiries can be directed to the corresponding authors.

## Supplementary Materials

The following supporting information can be downloaded at: https://www.mdpi.com/article/10.3390/ijms27167218/s1. References [56,57,58] are cited in the Supplementary Materials.
